# Is the ‘blue’ colour convention for inhaled reliever medications important? A UK-based survey of healthcare professionals and patients with airways disease

**DOI:** 10.1038/npjpcrm.2016.81

**Published:** 2016-11-03

**Authors:** Monica Fletcher, Jane Scullion, John White, Bronwen Thompson, Toby Capstick

**Affiliations:** 1 Education for Health, Warwick, UK; 2 Respiratory Department, University Hospitals of Leicester, Leicester, UK; 3 Respiratory Department, York NHSFT, York, UK; 4 UK Inhaler Group, Leeds, UK; 5 Pharmacy Department, Leeds Teaching Hospitals NHS Trust, Warwick, UK

## Abstract

In many countries, short-acting β_2_-agonist inhalers have traditionally been coloured blue. This inhaled therapy has also conventionally been known as a ‘reliever’ by patients and healthcare professionals (HCPs), in comparison with ‘preventer’ medications (inhaled steroids). With the rapidly changing market in inhaled therapy for COPD and asthma and growing numbers of devices, there has been some concern that the erosion of traditional colour conventions is leading to patients (and HCPs) becoming confused about the role of different therapies. In order to assess whether there was concern over the perceived changing colour conventions, the UK Inhaler Group carried out a large online survey of patients and HCPs. The aim was to determine how patients and HCPS identify and describe inhaled drugs, and how this might impact on use of medicines and safety. The results of the survey highlighted the importance of the term ‘blue inhaler’ for patients with only 11.3% never referring to the colour when referring to their inhaler. For HCPs, 95% felt colour conventions were important when referring to reliever medication. In addition, HCPs appear to refer to inhalers mainly by colour when talking to patients. Our conclusions were that the concept of a ‘blue inhaler’ remains important to patients and healthcare professionals. These results add to the debate about the need to formalise the colour coding of inhaled therapies, in particular using the colour blue for inhalers for rapid relief of symptoms, as this convention may be an important measure and contributor to patient safety. Our survey should provide impetus for all interested parties to discuss and agree a formal industry-wide approach to colour coding of inhaled therapies for the benefit of patients and carers and HCPs.

## Introduction

Inhalation is the preferred delivery route for asthma and chronic obstructive pulmonary disease (COPD) medications and therefore has a crucial role in the management of these common respiratory conditions.^1^

In the UK, parts of Europe and other countries including Canada, Australia and New Zealand it is common practice to refer to short-acting β_2_-agonist inhalers (‘reliever’ medications), such as salbutamol, as ‘blue inhalers’ based on their colour. The word ‘reliever’ has become a common terminology for patients and healthcare professionals (HCPs) alike as these inhalers provide rapid relief of symptoms because of their mode of action.

The evidence that patients and HCPs frequently refer to inhalers by colour rather than by the brand or generic name is largely anecdotal.^2^ However, the usual practice of using colours to distinguish mode of action has enabled patients and HCPs to more easily differentiate reliever medications from other classes of inhaled therapies such as inhaled steroids (the preventers). When determining disease control and giving advice on management, healthcare professionals often refer to inhalers by their colour.^3^

There is no formal agreement between manufacturers about a colour convention for the inhalers they manufacture. Further, no relevant legislation is issued by regulators of medicines including the Medicines and Healthcare products Regulatory Agency (MHRA) in the UK and European Medicines Agency.^4^ Nevertheless, this informal convention has been the accepted practice for several decades,^5,6^ and is frequently quoted by patient organisations in the UK such as Asthma UK,^7^ as well as in other countries such as Australia (Asthma Australia).^8^

With the introduction of a wider range of new inhaler devices, classes of drugs, compounds and combinations over recent years, the inhaler landscape has become increasingly complex for both HCPs and patients. In addition, as the colour coding of inhalers is no longer consistent, it has the potential to cause unnecessary confusion and safety issues. In the UK currently 18 licensed medications are available as either single or multiconstituent inhaled preparations for use in the treatment of asthma and COPD.^9^ An increasing array of colours is now being used by pharmaceutical companies to differentiate the treatments within their own product range, rather than demonstrating any conformity to specific colour conventions, perhaps apart from the choice of blue for relievers.

In the last two years two inhalers with significant amounts of blue colouring on them have been licensed for use in the UK^10,11^ although neither product was licensed for the emergency relief of symptoms. Issues regarding patient safety were expressed by the respiratory community, which was concerned that this would have misled patients resulting in inappropriate use with attendant risk. After significant lobbying from patient and professional organisations, both companies changed to a colour other than blue for their inhaler devices.^12,13^

The UK Inhaler Group (UKIG), formed of 11 UK-based patient groups, professional organisations and societies interested in respiratory issues, sought to establish whether the blue colour convention for reliever medications is an important issue for both patients and HCPs. UKIG therefore undertook a survey in September 2015 to establish the views of people living with asthma and/or COPD and HCPs.

## Results

### Healthcare professional survey

A total of 596 individuals responded to the HCP survey. Respondents identified their professional background as follows: 39% nurses; 17% doctors (GPs or hospital doctors); 14% respiratory physiologists; 13% pharmacists; and 8% other allied health professionals such as physiotherapists, healthcare assistants and pharmacy assistants; 9% did not specify their profession.

The most commonly reported method for healthcare professionals in identifying inhalers was to refer to their colour, with 45.6% reporting this to be their usual practice, 21.2% who reported that this was often their practice, whereas only 0.9% reported never referring to inhalers by colour. This is in contrast to 19.0% whose usual practice was to use brand names, and 13.6% whose usual practice was to use generic names (Table 1). Overall, it was apparent that most HCPs use a mixture of methods (colour, brand name and generic name) to refer to inhalers, but the majority of HCPs (86.8%) reported that they usually or often described the colour to refer to inhalers to patients. Two-thirds (66.7%) of respondents also reported that they usually or often also used the brand name to refer to inhaled medicines, but less than half (45.4%) used the generic name.

A total of 521 respondents (89%) said they used the terms relievers and preventers when talking to patients about their medications. In addition, nearly 95% of the HCPs felt colour convention to be important (Table 2). In fact, 87% felt it would be helpful if all relievers were coloured blue, and if a colour convention was to be retained it should be that all blue inhalers should be relievers. Only 11% felt that the broadening of the range of available inhaled medications had eroded the traditional colour conventions and agreed that the use of such colour conventions was now meaningless.

When asked whether they would like to add any further comments about the traditional colour conventions for inhalers, there were 241 additional comments from HCPs. The findings of the content analysis of respondents comments, together with representative quotations for each relevant emergent theme and cluster, are provided in Table 3. The most frequently encountered themes included the importance of maintaining the blue colour coding of reliever inhalers, recognising that it could be difficult but desirable to do this for other drug classes, and the difficulty for patients to remember drug names.

### Patient survey

A total of 2,517 people with airways disease responded to the patient survey. Of these, 390 were excluded from the survey because they either did not specify what lung disease they had (*n*=303), they had another lung disease other than asthma or COPD (*n*=77) or they did not know their diagnosis (*n*=10). Of the 2,127 patients included in the study, 82.5% (1,755) had asthma, 160 (7.5%) had asthma and COPD and 212 (10%) had COPD.

A total of 1269 (60%) patients said they used the terms reliever and preventer when talking about their inhalers. Patients most frequently reported that they usually identified their inhaled medication by describing either the colour (49.8%) or brand name (35.4%), rather than by the generic drug name (18.7%; Table 4). Only 11.3% of patients never referred to their inhaler by colour when talking to a HCP, whereas nearly 1 in 5 (18.2%) never used the brand name and more than a quarter (28.6%) never used the generic name. Patients with asthma and COPD were similar in their likelihood of using colour to refer to their inhalers. However, COPD patients were more likely than those with asthma to also usually refer to their inhalers by brand name (48.9% vs. 35.1%, *P*<0.005) or generic name (34.1% vs. 16.7%, *P*<0.005).

There are several responses indicating that colour has an important role in patients’ understanding of their medications (Table 5). Two most notably are that almost 80% of patients (1,674) strongly agreed/agreed that they like knowing that blue inhalers are there to relieve symptoms and 54% (Q3e) would be concerned if a preventative inhaler was mainly blue. Responses to two questions (Q3h and Q3i) suggest that it would not be easy to discontinue the practice of referring to inhalers by colour. Almost 90% felt if there was one colour tradition that should be kept, it would be that blue inhalers are to relieve symptoms.

However, about half the respondents felt that it was more important to understand the role of the medications contained within the inhaler rather than the colour (Q2).

Patients with asthma were more likely than those with COPD to have strong opinions about the colour of inhalers, although a higher proportion of patients in both groups agreed that colour of inhalers was an important issue. In contrast, patients with COPD were more likely than those with asthma to have stronger opinions about the need to understand what each medicine was for and know the name of their medicines.

Patients provided further comments on the use of inhalers. The findings of the content analysis of respondents’ comments, together with representative quotations for each relevant emergent theme and cluster, are provided in Table 6. The most frequently encountered themes again included the importance of maintaining colour conventions for inhalers, in particular, restricting the use of blue colour for reliever inhalers and how this aids not only the patient but also family, friends, colleagues and school teachers. A total of 88 respondents reported that they were not aware of an association prior to completing this survey, although at least seven of these were patients in countries such as USA and Brazil where no such colour convention exists. A total of 30 respondents suggested alternative colours for inhalers, with some suggesting on more consistent colours for preventer inhalers and six suggesting that red would be more suitable as a colour to emphasise the emergency role of reliever inhalers.

## Discussion

### Main findings

Although recognising the limitations of a survey approach, this survey provides a message that patients and HCPs like to use the term ‘blue inhaler’ and that clinicians in this study predominantly refer to inhalers by colour when talking to patients. At the same time there was some indication that patients used a mix of colour and the brand name of the medicine in the inhaler, particularly those with COPD.

The informal colour convention for inhalers appears to be helpful in aiding communication between HCPs and patients. It helps patients understand the different role of their inhalers, and how they should be taken, which is particularly useful for discussing the difference between relievers and preventers. With more inhalers coming onto the market, the choice of treatments is going to continue to widen. It would appear that the majority favour keeping the colour blue for relievers.

Without this colour convention, patient education could become more complex and could cause confusion. This could be the case particularly for those who are not HCPs, who have responsibility to care for children and adults with asthma, such as school teachers and social care workers, and indeed parents themselves. This raises concerns regarding patient safety.

Colour coding has been used in a wide range of heterogeneous healthcare settings in a multitude of ways such as syringe labelling in anaesthetics.^14–16^ In developing countries colour coding in healthcare is an important tool to promote the extension of quality assured healthcare services provided by outreach workers and unqualified healthcare providers.^17^

Any change to the colour convention would require a major re-education of HCPs, and more importantly, patients and their carers. However, a potential amendment is the use of a system of embedding standardised coloured circles or dots onto the casing of the inhaler.^18^ This would allow for combinations of medications and their actions to be easily explained to patients. The authors of these recommendations, however, stress that a blue dot should be universal for a short-acting beta agonist.

An alternative view suggests that differentiating inhalers by colour may cause problems to some patients because of colour blindness. It is estimated that 8% of men and 0.5% of women worldwide have a colour vision deficiency.^19^ However, illiteracy is a far bigger problem worldwide and an inability to perceive blue light (tritanopia) is an extremely rare form of colour vision impairment affecting as few as 1 in 30,000–50,000 and therefore is less of an issue regarding the blue inhaler, but nevertheless a consideration.^20^

Whatever future decisions are taken about the ways of distinguishing inhalers, patient safety is of paramount importance. There are therefore major implications for licensing authorities and groups focused on this aspect of patient safety.

### Interpretation of findings in relation to previously published work

There are little other published data addressing this subject, and it is remarkable that studies designed to clarify this debate have not taken place, despite the expansion of inhaled therapies for airways disease.

### Strengths and limitations of this study

This study is a survey of the views of patients and HCPs and not a clinical trial; therefore, our findings will need confirmation in further studies. Demographic data such as age, gender and age when diagnosed were not collected, but would have been useful in the interpretation and understanding of the results. The questionnaires were self-completed, so we are unable to verify that all respondents have the conditions asthma and/or COPD, as in any survey. In our view, this is counter balanced by the large number of responses we were able to obtain using this survey method. As we used a web link to distribute the survey via multiple access points, it was not possible to prevent duplicate responses or to determine if they occurred, nor could we gather responses from those without access to electronic media or those with reading difficulties. Another limitation was that it was not possible to determine how many individuals accessed the survey and then subsequently declined to participate, so a response rate cannot be estimated: nor did it include those without access to electronic media or those with reading difficulties.

This survey was designed specifically to target the opinions of UK patients and HCPs using social media and websites to promote the survey; however, as these access points are also available to people outside of the UK, we found that some patients from outside the UK also completed the survey. As a colour convention may not be the norm in other countries, this could potentially have affected our results. As we did not collect the data on country of residence, we cannot be certain about how many responders were non-UK residents. Review of individual comments alerted us to the fact that at least seven patients lived or have lived in other countries, but we cannot be certain whether other responders were also non-UK residents. Consequently, we did not correct the data for the seven patients we knew to be non-UK residents, as this could have excluded non-UK data in an incomplete manner. As our invitation letter specified that this was a UK survey, we believe that there will have been a negligible number of respondents from outside of the UK, and consequently would not have adversely affected our results.

We accept that there are disadvantages to surveys in that respondents may not feel encouraged to provide accurate, honest answers and that surveys with closed-ended questions may have a lower validity rate than other question types. We recognise that answers rely on interpretation by the respondent and that it will always be the interested parties that respond to surveys. We also acknowledge that HCPs with an interest in colour conventions may be more likely to have taken part although this may not apply to the large numbers of patients who participated.

Although it was not our intention to introduce bias into the study, it could be considered that there was bias towards the 'usefulness of colour conventions' because of the questions focusing on colour. This focus could be seen to narrow the research question being asked. Further research concentrating on the question construction would assist with generalisability of our findings.

However, these points can be mitigated by the fact that surveys are relatively easy to administer, are cost-effective and capable of collecting data from a large number of respondents. They give the flexibility to ask a number of questions and can be analysed with appropriate software.

There was also a strong theme in about half of our respondents who felt that it was more important to understand the role of the medications contained within the inhaler rather than the colour.

Of interest only 11% felt that the broadening of the range of available inhaled medications had eroded the traditional colour conventions and agreed that the use of such colour conventions was now meaningless (statement 11). However whether this question was answered on the grounds of the many coloured combinations inhalers is unclear as it seems incongruent with former statements.

### Implications for future research, policy and practice

As the limitations previously identified in these results may not be representative, these surveys appear to highlight that inhaler colour is important and that there is a desire from HCPs and patients alike that the blue convention is maintained for patient safety issues. In terms of safety and governance until there is a shift away from current practice it appears implicit that colour coding should be standardised and reinforced.

It is inevitable that increasing numbers of inhalers will be introduced into the already complex environment which will no doubt widen the range of colours of inhalers. This may be more important for HCPs than for individual patients, who usually only have 1–3 inhalers.

There appears a need to take on board the views of clinicians and patients when considering inhaler device colour conventions. Colour needs to be given greater consideration by both pharmaceutical companies and licensing authorities to focus on the safety of patients when designing inhalers and selecting the colour.

HCPs also need to consider how they discuss inhalers with patients in order that patients understand how to use different types of inhalers and get maximum benefit from them. They should also recognise that many patients, particularly those with COPD, may also value provision of other descriptive information about their inhaled medicine, including the mode of action and generic/brand name.

### Conclusion

The importance of blue as the emergency inhaler is greater in asthma than in COPD, but understanding the role of different inhalers is still important in COPD to aid compliance, so that patients and NHS get best value from their treatments.

Consistency of colouring of inhalers should reduce the likelihood of error and may help to reduce the risk of asthma deaths; however, there is currently a lack of standardisation. This study confirms that clinicians are systematically using colours to refer to inhalers in discussions with their patients, and patients do the same. Of all the informal colour conventions, the use of blue for inhalers to be used for rapid relief of symptoms is most commonly adhered to and therefore of greatest importance to protect, particularly given the need for accurate use in acute situations. The ‘convention’ is still widely used in the UK and the results of this study suggest that, certainly at least in the short-to-medium term, this should be retained. Formalisation of this convention should be agreed, to promote patient safety, so that in future it would not be possible for a blue inhaler to obtain a license unless it is a reliever, and conversely that an inhaler that is not for rapid symptom relief will not be licensed if it is blue.

As new inhaled medications with different mechanisms of action will undoubtedly come to market in the future, this study suggests that the issue will need to be openly debated and that there should be widespread consultation with HCPs, patients, the pharmaceutical industry and those with the power to enforce legislative changes. It is not desirable or appropriate and may be costly for individual companies to make unilateral decisions, which could affect both patient safety and also adherence rates, and has been expressed as a concern by healthcare professionals.^21^

Colour conventions across all inhaler device classes may now have practical shortcomings as the number and types of inhalers have greatly expanded and will undoubtedly continue to do so. However, retaining the blue convention for reliever medications is still in our hands if we wish to preserve and indeed strengthen this on the grounds of patent safety.

## Materials and methods

An overarching online self-reported questionnaire was developed by a multi-professional group of HCPs and patient representatives of groups within UKIG. Two versions were then created, using slightly different terminology for people with asthma and COPD and their carers, and HCPs. Each was piloted by five patients and five HCPs in order to assess the appropriateness of the terminology used, test the survey instructions and to establish the time required to complete the survey. As a result two questions were removed entirely and some minor adjustments were made to the instructions and terminology.

Having given consideration to the required response rates, survey design, cost implications and timeframes, the decision was made to administer the survey via the internet, using SurveyMonkey (SurveyMonkey, Palo Alto, CA, USA [www.surveymonkey.com]). SurveyMonkey has been successfully used in published healthcare research and is a useful tool when widespread geographical coverage is desired.^22^ The results of the surveys were analysed using the software available from SurveyMonkey.

During a 4-week period, the surveys were made available to patients with a diagnosis of asthma and/or COPD and a range of HCPs from around the UK. The two surveys ran in parallel from 2 September 2015 to 5 October 2015. A generic website link for the survey was created, and the link was distributed in several ways including sending an email with the link to potential respondents on existing databases, placing the survey link on each of the UK-based organisations’ websites and using social media sites such as Twitter and LinkedIn. This methodology allowed for confidentiality for respondents and widespread awareness of the survey.

The invitation letter contained information about the survey as well as the web link that potential participants needed in order to gain access to it. The letter explained that UKIG was a group of organisations formed with the common desire to ensure that patients and the NHS get the maximum benefit from inhaled medications; of particular interest were the views on the colour of inhalers. Informed consent was assumed by the fact that all participants had to voluntarily access the web link to complete the survey. Although the surveys were made available through a variety of sources, it was requested that potential respondents completed it only once.

An exploratory analysis, using Chi-square statistical testing, was performed to investigate potential differences in opinions on the colour of inhalers between groups of patients with a diagnosis of asthma or COPD. No sample size calculation was performed as this was purely an exercise to investigate trends in opinions and not intended to be a definitive analysis.

A content analysis of responders’ comments was used to code the qualitative data into themes and categories, to evaluate the emergent topics that were highlighted by patients and healthcare professionals. This analysis was performed by one author (T.C.) and reviewed by all other reviewers in order to agree that the final categorisations were accurate.

## Funding

The authors declare that no funding was received.

## Figures and Tables

**Table 1:** How healthcare professionals usually refer to inhalers when talking to patients

	*Usually*, n *(%)*	*Often*, n *(%)*	*Sometimes*, n *(%)*	*Never*, n *(%)*	*Total*, n
By colour (e.g., ‘Use your blue inhaler if your symptoms get worse’)	268 (45.6%)	242 (41.2%)	73 (12.4%)	6 (0.9%)	588
By the brand name (e.g., Ventolin, Flixotide, Symbicort)	109 (19.0%)	274 (47.7%)	174 (30.3%)	17 (3.0%)	574
By the generic name (e.g., salbutamol, salmeterol, budesonide)	77 (13.6%)	180 (31.8%)	277 (48.9%)	32 (5.7%)	566

**Table 2:** Healthcare professionals’ responses to questions in the survey

	*Strongly agree /agree*, n *(%)*	*Neither agree nor disagree*, n *(%)*	*Disagree/strongly disagree*, n *(%)*	*Not applicable*, n *(%)*	*Total responders*, n
Colour conventions for inhalers are helpful	527 (94.8%)	20 (3.6%)	9 (1.6%)	0 (0%)	556
Colour conventions are not that important—patients need to understand the role of each medication, whatever their colour	168 (30.4%)	142 (25.7%)	240 (43.5%)	2 (0.36%)	552
It is helpful if all blue inhalers are relievers and inhalers of other colours are preventers	483 (87%)	51 (9.2%)	21 (3.8%)	0 (0%)	555
It would be easy to stop talking about inhalers by colour and use the words ‘preventer’ or ‘reliever’ instead	135 (24.4%)	161 (29%)	257 (46.5%)	0 (0%)	553
I would have concerns about safety if a blue inhaler were to contain a preventer treatment	481 (87%)	39 (7.1%)	33 (6%)	0 (0%)	553
Talking about inhalers by colour helps me to explain the role of different inhalers so that patients use them properly	449 (81%)	76 (13.7%)	27 (4.9%)	3 (0.4%)	554
Colour conventions are more important for inhalers than for other medicines, such as tablets	392 (71%)	117 (21.2%)	42 (7.6%)	1 (0.2%)	552
Using the words ‘reliever’ and ‘preventer’ helps me to explain the role of different inhalers so that patients use them properly	486 (88%)	46 (8.3%)	18 (3.3%)	2 (0.36%)	552
It would be easy for me to stop talking about inhalers by colour and use the proper name instead (e.g., Ventolin)	88 (15.9%)	173 (31.3%)	284 (51.4%)	8 (1.5%)	553
If there is one colour tradition worth retaining, it is that blue inhalers are for relieving symptoms	497 (89.7%)	43 (7.8%)	14 (2.5%)	0 (0%)	554
The introduction of combination inhalers and the long acting β_2_-agonist (LABA) and long acting muscarinic antagonist (LAMA) have eroded colour conventions from the days of just blue and brown inhalers, so colour conventions are now meaningless and not worth trying to preserve	63 (11.3%)	164 (30%)	317 (57%)	12 (2.2%)	556

**Table 3:** How healthcare Professionals refer to inhalers, and selected quotations

*Relevant Emergent Themes (no. of HCPs reporting)*	*Relevant quotations to illustrate theme*
*Colour of inhalers*	
Importance of blue colour (168)	‘Colour is useful particularly when explaining to people with learning disabilities, dementia, those who can't read and young children’ ‘I think having the colour codes is a huge safety net that should never be changed. In an emergency it is lifesaving.’
Colour scheme for other classes of inhaled therapy (40)	‘Combination inhalers should show both colours I.e. if all LABA were blue, all LAMA were green and combination inhalers had both colours on.’ ‘(it) would be great to also be able to use colours to indicate types of combinations—isnt [*sic*] just patients who dont [*sic*] know!’
Difficulties in achieving colour conventions (30)	‘I just think we need to be preparing ourselves for getting away from colour connections as with the influx of new devices and more to come it is probably safer to talk about relivever [*sic*], preventers, combinations etc.’ ‘Perhaps a case of closing the stable door after the horse has bolted on this one but would have been good to establish a convention (relievers, LABAs, LAMAs, ICS, etc) whilst the opportunity was available.’
Vision problems (5)	‘Colour conventions do not work if your patient is blind or partially sighted.’ ‘Take colour blindness into account when considering colours for inhalers.’
	
*Nomenclature*	
Using drug name (22)	‘I only use the colour as an adjunct. I also say the brand name and describe the job it does.’ ‘Most patients are aware of the proper names of their inhalers, but I find that the more elderly find it easier to refer to them by colour’
Difficulty with drug names (26)	‘I find quite a lot of patients do not know the name or nature of their inhalers and only refer to them by colour—this is the only way I can then identify what regime they are on’ ‘Not all patients understand medication names, they are often long and complicated. Also, the use of generic names as well as brand names can make it further complicating. We need colour AND names for differing needs of patients.’
	
*Other methods to refer to inhalers*	
Mode of action (34)	‘Patients need to be made more aware of relievers and preventers’ ‘…I think a better system could be a wording on the product of ‘RELIEVER’ and ‘PREVENTER’ in a standard font, in a box, and possibly coloured!’
Importance to combine methods to educate patients (25)	‘Colour convention is particularly helpful where patients have reduced capacity of understanding. I would also use generic/brand names/reliever/ preventer terms etc if possible/according to each patients capability—without confusing them!!’ ‘I like to use different terms and fit them to suit my patients understanding as some work better with names and I think they should show the name. However most people don't know the name but remember the colour more easily.’
	
*Patient factors*	
Patient comprehension and education (32)	‘If patient has dementia for example the traditional blue colour is much easier for them to remember as it has been the same for years.’ ‘Poor health literacy for many patients means that terms we think are simple such as reliever and preventer can be confused.’ ‘I think patients are more adept at understanding than we give them credit for and actually taking time to talk and provide education in a consistent manner is more important.’ ‘Educating the patient about their treatment is the most important over colour coordinating inhalers as a poorly educated patient will still get confused with colours’

**Table 4:** How patients usually refer to inhalers when talking with a healthcare professional

*Patient group*	*Usually*, n *(%)*	*Often*, n *(%)*	*Sometimes*, n *(%)*	*Never*, n *(%)*	*Total*, n	P *value*
*By colour (e.g., ‘I use my blue inhaler if my symptoms get worse’)*						
Asthma (*n*=1,755)	813 (50.7%)	286 (17.8%)	332 (20.7%)	172 (10.7%)	1,603	0.86
COPD (*n*=212)	79 (48.2%)	32 (19.5%)	33 (20.1%)	20 (12.2%)	164	
All (*n*=2,127)	943 (49.8%)	309 (17.9%)	397 (21.0%)	214 (11.3%)	1893	
						
*By the brand name (e.g., Ventolin, Flixotide, Symbicort)*						
Asthma (*n*=1,755)	513 (35.1%)	313 (19.6%)	458 (28.7%)	313 (19.6%)	1,597	<0.005
COPD (*n*=212)	80 (48.8%)	24 (14.6%)	40 (24.4%)	20 (12.2%)	164	
All (*n*=2,127)	673 (35.4%)	360 (18.9%)	523 (27.5%)	346 (18.2%)	1902	
						
*By the medicine inside (generic name) (e.g., salbutamol, salmeterol, budesonide)*						
Asthma (*n*=1,755)	250 (16.7%)	205 (13.7%)	591 (39.4%)	454 (30.3%)	1,500	<0.005
COPD (*n*=212)	45 (34.1%)	17 (12.9%)	45 (34.1)	25 (18.9%)	132	
All (*n*=2,127)	328 (18.7%)	247 (14.1%)	679 (38.7%)	502 (28.6%)	1757	

**Table 5:** Patients’ responses to questions in the survey

*Answer options*	*Patient group*	*Strongly agree /agree, n (%)*	*Neither agree nor disagree, n (%)*	*Disagree/strongly disagree, n (%)*	*Not applicable, n (%)*	*Total responders, n*	P *value*
a) Talking about inhalers by their colour is helpful	Asthma (*n*=1,755)	1375 (79%)	241 (14%)	126 (7%)	3 (0%)	1,745	0.005
	COPD (*n*=211)	132 (65%)	47 (23%)	21 (10%)	3 (1%)	203	
	All (*n*=2,127)	1598 (76%)	330 (16%)	165 (8%)	7 (0%)	2,100	
b) The colour of inhalers is not important—it is more important to understand what the medicine inside is for	Asthma (*n*=1,755)	862 (50%)	441 (26%)	401 (23%)	13 (1%)	1,717	0.005
	COPD (*n*=211)	132 (66%)	34 (17%)	35 (17%)	(0%)	201	
	All (*n*=2,127)	1089 (53%)	505 (24%)	462 (22%)	13 (1%)	2,069	
c) I like knowing that blue inhalers are to relieve symptoms and inhalers of other colours are to prevent flare ups	Asthma (*n*=1,755)	1413 (81%)	232 (13%)	87 (5%)	14 (1%)	1,746	<0.005
	COPD (*n*=211)	152 (74%)	40 (19%)	8 (4%)	6 (3%)	206	
	All (*n*=2,127)	1674 (79%)	305 (14%)	106 (5%)	21 (1%)	2,106	
d) It would be easy to stop talking about inhalers by colour and use the words 'preventer' or 'reliever' instead	Asthma (*n*=1,755)	478 (28%)	539 (31%)	683 (39%)	32 (2%)	1,732	0.05
	COPD (*n*=211)	70 (34%)	46 (22%)	86 (42%)	3 (1%)	205	
	All (*n*=2,127)	609 (29%)	630 (30%)	812 (39%)	39 (2%)	2,090	
e) I would be concerned if an inhaler to prevent a flare up was mainly blue	Asthma (*n*=1,755)	976 (56%)	434 (25%)	290 (17%)	33 (2%)	1,733	<0.005
	COPD (*n*=211)	88 (43%)	76 (37%)	37 (18%)	3 (1%)	204	
	All (*n*=2,127)	1129 (54%)	565 (27%)	353 (17%)	40 (2%)	2,087	
f) Talking about inhalers by colour helps me to understand the role of different inhalers so that I use them properly	Asthma (*n*=1,755)	1059 (61%)	405 (23%)	247 (14%)	27 (2%)	1,738	<0.005
	COPD (*n*=211)	101 (49%)	69 (34%)	29 (14%)	6 (3%)	205	
	All (*n*=2,127)	1233 (59%)	518 (25%)	305 (15%)	37 (2%)	2,093	
g) Colours are more important for inhalers than are for other medicines, such as tablets	Asthma (*n*=1,755)	1125 (65%)	401 (23%)	186 (11%)	15 (1%)	1,727	0.59
	COPD (*n*=211)	134 (65%)	42 (20%)	26 (13%)	3 (1%)	205	
	All (*n*=2,127)	1345 (65%)	475 (23%)	239 (11%)	20 (1%)	2,079	
h) Using the words 'reliever' and 'preventer' helps me to understand the role of different inhalers so I use them properly	Asthma (*n*=1,755)	1068 (62%)	412 (24%)	239 (14%)	16 (1%)	1,735	<0.005
	COPD (*n*=211)	110 (54%)	44 (22%)	45 (22%)	5 (2%)	204	
	All (*n*=2,127)	1261 (60%)	498 (24%)	307 (15%)	23 (1%)	2,089	
i) It would be easy to stop talking about inhalers by colour and use the proper name instead (e.g., Ventolin)	Asthma (*n*=1,755)	519 (30%)	502 (29%)	676 (39%)	36 (2%)	1,733	<0.05
	COPD (*n*=211)	84 (41%)	52 (25%)	70 (34%)	1 (0%)	207	
	All (*n*=2,127)	673 (32%)	591 (28%)	786 (38%)	40 (2%)	2,090	
j) If there is one colour tradition that should be kept, it would be that blue inhalers are to relieve symptoms	Asthma (*n*=1,755)	1545 (89%)	146 (8%)	48 (3%)	3 (0%)	1,742	<0.005
	COPD (*n*=211)	172 (84%)	27 (13%)	3 (1%)	3 (1%)	205	
	All (*n*=2,127)	1839 (88%)	196 (9%)	55 (3%)	8 (0%)	2,098	

**Table 6:** How patients refer to inhalers, and selected quotations

*Relevant Emergent Themes (no. of Patients reporting)*	*Relevant quotations to illustrate theme*
*Colour of inhalers*	
Importance of blue colour (196)	‘I think it is useful that blue is synonymous with reliever inhalers, as asthmatics and many non-asthmatics know what type of inhaler that is and in an emergency it's good to just be able to call it by its colour rather than remember the brand name.’ ‘My 7 year old knows the blue one is for when I am having an attack. It's useful to tell people I need the blue one and quicker therefore to get the medicine I need.’
Impact of colour association (total 273)	‘Blue has always been known as reliever for many asthmatics and brown the preventer, but there are other inhalers which are preventer/reliever combos such as seretide and symbicort and long acting relievers which come in green coloured inhalers’ ‘The different colours are very helpful for my asthma as each colour helps me to associate what each inhalers job is. If they were all the same colour I don't think I would cope!’ ‘People often associate the colour with a 'reliever' or 'preventer', people associate and remember colours easier than names.’ ‘I think during an attack the colour recognition is extremely important. I also feel it is important to understand the medication within your inhaler and its role in treating asthma however the colour in a serious attack is extremely important’ ‘There are too many different colours and...it is much more important to know what they are by brand name such as salbutamol or ventolin’
Alternative colour suggestions (30)	‘It would make sense if the relievers were of a red colour. Relievers are used to relieve symptoms of possible attacks; red indicates urgency!.’ ‘The well known colours for preventers could be changed but please don't change the blue relievers.’
Colour scheme for other classes of inhaled therapy (31)	‘The preventer inhalers' shades can be too similar (brown, brown-ish, brown but more red... red)—I've seen this confuse asthmatics and specialist chest doctors alike.’ ‘With Symbicort SMART being used more and more often, it's making the use of the term ‘blue inhaler’ more difficult to transfer to other inhalers that aren't blue and for people to understand their purpose.’
Experience outside of the UK (7)	‘This isn't the case in all countries—e.g., in the USA some relievers are yellow/orange rather than blue, so it doesn't make sense to rely on colour rather than the actual name and function of the medication.’ ‘I've just started using Relvar Ellipta and I struggle to explain to my husband which one I need because they are all blue! ‘
Vision (34)	‘I am visually impaired and rely very much on the colour of my inhalers. It's also great to be able to say go my kids please fetch my blue inhaler for example and know they get the right one.’ ‘The colours are great, though the shape is more important when you take into consideration colour blindness and actual blindness.’
No association (88)	‘Have never even considered the colours as I know the names, what each is prescribed for and the dosage/times to use.’ ‘I hadn't even realised that reliever inhalers were blue and preventers other colours…and I've been taking asthma meds for over twenty years...’
Impact on friends, family, carers, colleagues (156)	‘I think blue is helpful for relievers for school staff when helping children to use medication as it is currently a universal idea.’ ‘it's also easier for the people I am with to know, i.e., family and partners know to grab the blue inhaler when I am having an attack’
Resistance to change (152)	‘Keep the blue colour for reliever. Everyone knows the blue inhaler is the one needed during an asthma attack.’
Ease of understanding (153)	‘Easy to understand that blue is the reliever inhaler and other colours preventers’ ‘The different colours are very helpful for my asthma as each colour helps me to associate what each inhalers job is. If they were all the same colour I don't think I would cope!’
	
*Nomenclature*	
Using drug name (51)	‘Lucky for me I understand mine, and are happy to use the name, although I sometimes don't pronounce it properly but most clinician's know what I mean, & understand me.’ ‘Understanding your different medicines by name and intention is...important.’
Difficulty with drug names (53)	‘Most inhalers have unpronucable names..if referring to these inhalers by colour its [SIC] easier for the patient to understand’ ‘For me and the people I have talked to that have asthma it is a lot easier to remember colours than the complicated names. ‘
	
*Other methods to refer to inhalers*	
Mode of action (50)	‘...it is important to know what each one is called medically and what it does not just by colour.’ ‘It is very important to understand the inhalers as ‘preventers’ and ‘relievers’, for me these have been explained fully by health professionals, and the association of the colours goes hand in hand with that.’
Importance to combine methods to educate patients (14)	‘...people should be educated on their meds and not rely on colours.’ ‘I think for asthma patients, it is much more important to understand what medicine is contained inside the inhaler and how that medicine works and is to be used, rather than simply referring to the inhaler as by colour.’
Shape of the inhaler (17)	‘The colours are great, though the shape is more important when you take into consideration colour blindness and actual blindness.’
	
*Patient factors*	
Patient comprehension and education (149)	‘It's easier using colours especially for young children to understand which to use and when’ ‘Using the same colour overall for reliver inhalers is useful for both children and the elderly and also for people with learning difficulties’
